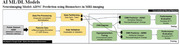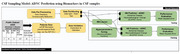# Beyond Images : Seeing the unseen for early detection of Alzheimer's Disease

**DOI:** 10.1002/alz70861_108065

**Published:** 2025-12-23

**Authors:** Shivam Joshi‐Awasthi

**Affiliations:** ^1^ North Penn High School, Lansdale, PA USA

## Abstract

**Background:**

Current methods of early detection of Alzheimer's Disease (AD) are not universally available across all geographies. An AI/DL based approach could accelerate the early detection of AD.

**Method:**

We investigated the feasibility of developing an AI/DL based model for early detection of AD using cross‐sectional (T‐1 MRI scans) and longitudinal (CSF) biomarkers. T1 MRI data, was used to develop a ML model to classify subjects with and without AD. We were able to disambiguate “healthy” vs late‐stage AD and our model can disambiguate “healthy” and “early onset AD”. Using CSF data, we developed a model to predict the progression rate of AD given age and a variety of other covariates. ML techniques were applied to i) T1 MRI imaging to classify subjects as “normal aging”, “early‐onset”, or “moderate AD” measured in terms of ADNC and ii) CSF biomarker data to predict ALSFRS‐r (Amyotrophic Lateral Sclerosis Functional Rating Scale) scores and monthly progression factor.

**Results:**

Accuracy and F1 metrics on the test/train dataset were 94.3% and 94.3% for Random Forests, 92.4% and 93.2% for Stochastic Gradient Descent, and 97% and 97% for CNNs. The CNN was able to differentiate early onset patients from normal aging with an accuracy of 94%. Using CSF data, the NN achieved a final MAE: 39.39/40.51; RMSE: 39.95/40.6 in training/testing respectively. Additionally, for evaluating ML methods as a predictive model for AD, we **performed meta‐analysis using the predicted ALSFRS‐r scores**, which calculated the time (in years) when patients might reach different points of AD progression (Normal Aging, Low/Moderate ADNC) thereby predicting the window of early detection and intervention for AD.

**Conclusion:**

Utilizing the outputs of the MRI‐imaging model and CSF sample model, we estimated approximately when a patient may enter stages of Low and Moderate ADNC, potentially helping reveal the otherwise ‘missed period of intervention’. This could result in faster treatments for patients still in a treatable phase of their disease, and provide a model for understanding disease progression overtime. Finally, computing a salience map we can potentially determine plaque, NFT, and other biomarkers’ locations, enabling higher treatment accuracy, thus more ‘personalized’ treatment for patients.